# ResearchRabbit

**DOI:** 10.29173/jchla29699

**Published:** 2023-08-01

**Authors:** Victoria Cole, Mish Boutet

**Affiliations:** 1 Research Librarian (Health Sciences) University of Ottawa; 2 Digital Literacy Librarian University of Ottawa

**Product:** ResearchRabbit

**URL:** https://www.researchrabbit.ai/

## Product Description

ResearchRabbit is a scholarly publication discovery tool supported by artificial intelligence (AI). It was developed in 2021 by a team of three in Seattle [1]. This tool lets users discover publications related to one or more seed publications with the help of visualization maps and lists of earlier, later, and similar publications. ResearchRabbit is designed to support the workflow of unstructured searching while providing a left-to-right trail from the original publication(s) through any selected authors or publications. These trails, which can run as deep as rabbit holes, suggest the origin of the tool’s name.

To start using ResearchRabbit, users first need to create an account. Then they need to create a collection and add at least one publication. The more publications that are added, the better ResearchRabbit can understand users’ interests and generate recommendations similar to the contents of the collection. Publications can be added either by uploading a RIS or BibTeX file or by using ResearchRabbit’s search, powered by PubMed, if users are searching the medical sciences, or Semantic Scholar, for any other subject area. While ResearchRabbit uses PubMed’s and Semantic Scholar’s search engines, the company claims its unique database of “100s of millions of academic articles” is second in size only to Google Scholar [2].

**Fig. 1:**
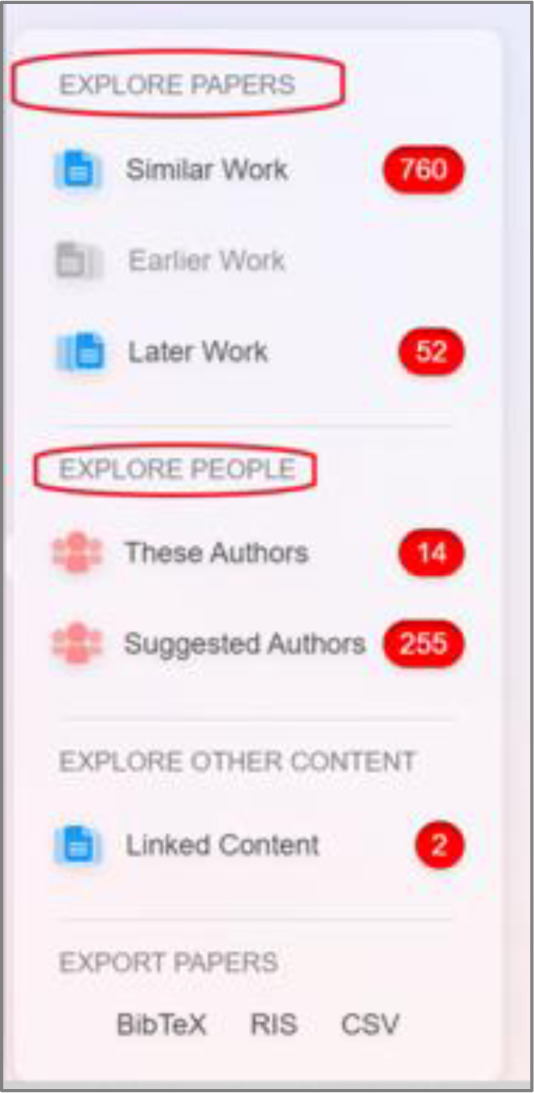
Different exploration modes

Once publications are in a collection, ResearchRabbit’s algorithm will begin generating recommendations. These recommendations can be explored through two modes: 1) by *Papers* that are *Similar work, Earlier work*, or *Later work* or 2) by *People* that provide additional publications that *These authors* or *Suggested authors* have published (Figure 1). These recommendations are depicted using visualization maps.

## Intended Users

ResearchRabbit is intended for any researcher conducting an unstructured publication search. ResearchRabbit can be used for supplementary searching to complement comprehensive database searches for users doing knowledge synthesis projects.

## Special Features

### Visualization maps

**Fig. 2:**
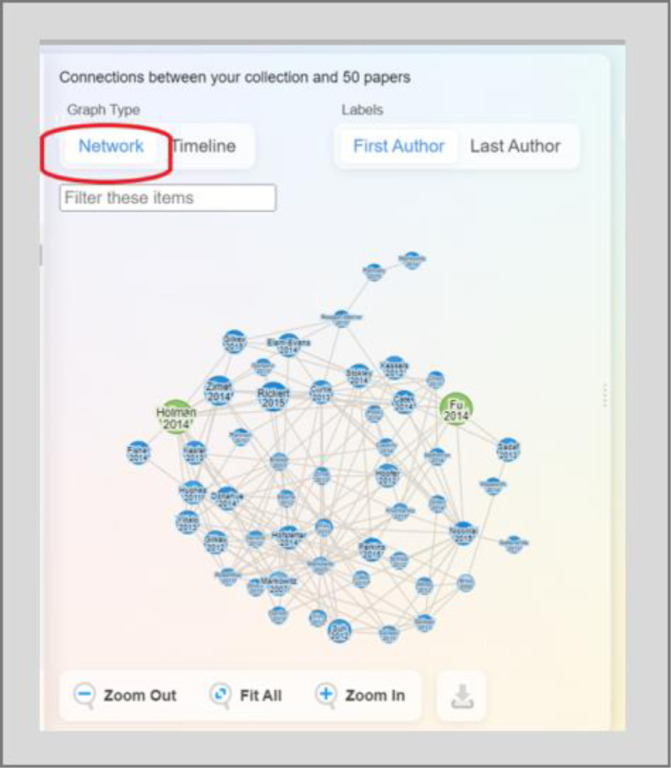
Visualization map in Network view

**Fig. 3:**
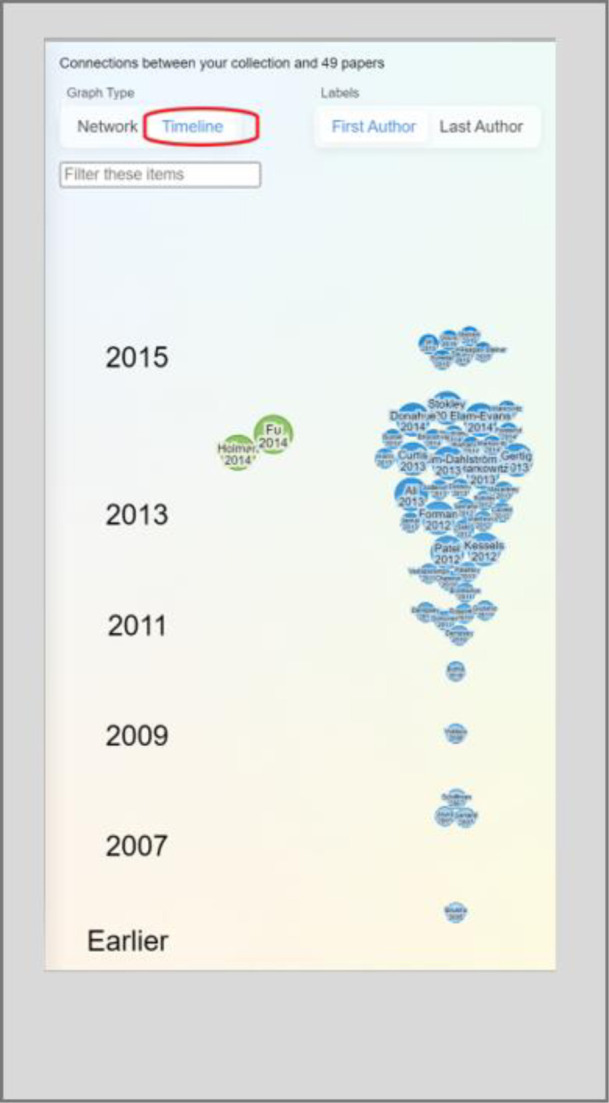
Visualization map in Timeline view

Publication recommendations are presented as visualization maps. There are two views for these maps. Network view allows users to see publications that are connected to one another (Figure 2). When choosing the options of earlier or later work, publication recommendations are primarily based on citations. Similar work relies more heavily on ResearchRabbit’s algorithm than on citations to generate recommendations. In ResearchRabbit’s FAQ, the team says little about the workings of the recommendation engine, briefly mentioning citation networks and “some additional magic” [2]. Timeline view plots publications by year, illustrating when the work was published in the field (Figure 3). In both views, each publication is represented by a node. Green nodes represent publications already in the user’s collection and blue nodes are not in the collection. The darker shades of blue represent more recent publications. Visualization maps can also depict networks of collaborating authors, each of whom is represented by a red node.

### Unstructured searching workflow

**Fig. 4:**
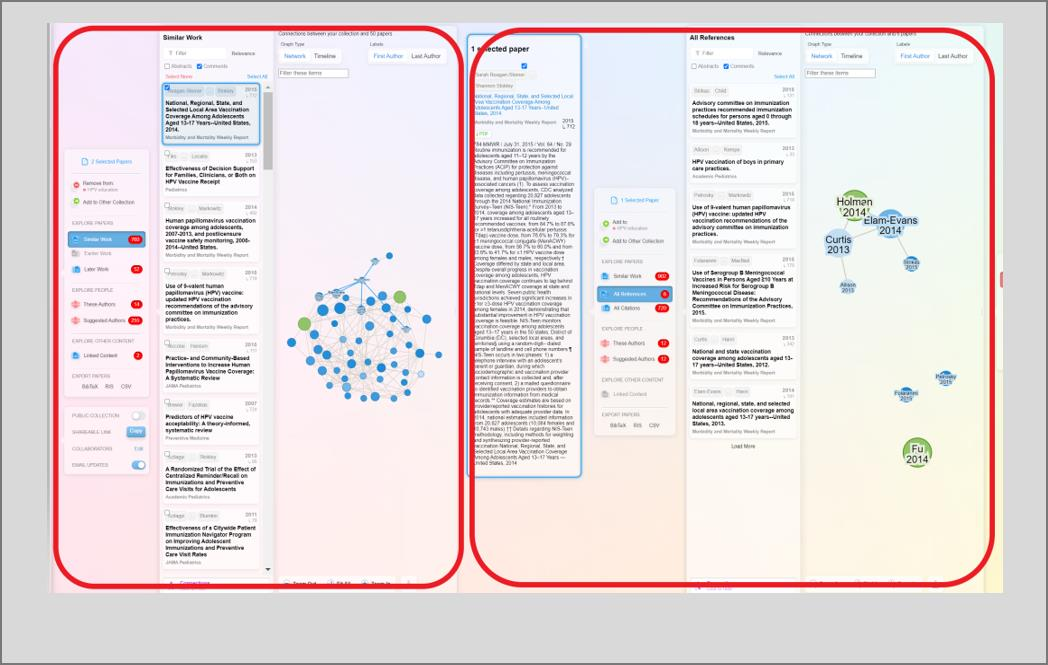
Each panel represents a new search

Searching for literature can be haphazard, specifically when users are not conducting searches for knowledge synthesis projects. For example, a user might start skimming for keywords in an article and then come across a prominent author in this research field, which can lead to wanting to know all the publications of this author and looking at the reference list for a newly discovered article of interest. A challenge with this type of searching is that it is possible to get lost down a rabbit hole of endless associated authors and citations. ResearchRabbit supports this workflow and creates a linear trail for wayfinding. A new panel opens to the right as a different type of search is conducted (Figure 4). To find your way back, scroll back to the left.

## Usability

ResearchRabbit is accessed via web browser without the need for additional software. The site is generally navigable by keyboard and with the aid of a screen reader. However, there are some navigation challenges, including many buttons and a high volume of condensed information presented by ResearchRabbit, which can make it difficult for users to know where to begin.

## Strengths

Visualization maps allow users to see connections between publications or authors they may not otherwise notice. For example, when exploring by authors, users can find research teams that they may not have been aware of. The Similar Works feature may help unearth related publications that would not be discovered with other search tools.

## Weaknesses

ResearchRabbit has many different features, which can be overwhelming to explore. A large initial time investment is required to learn the different features to use ResearchRabbit to its maximum potential.

ResearchRabbit currently limits exploration to a single linear path and leaves users to remember which branching paths of authors and citations they have already explored. The interface does not provide the means to save a rabbit hole for later exploration, nor does it provide a means for users to mark publications they have already come across, unless they are in one of the user’s collections.

The author visualization maps have some trouble with author disambiguation. It is not uncommon in ResearchRabbit for a single author to appear as two nodes in a collaboration network, each of which is associated with different publication and citation numbers.

## Cost/Value

At the time of publication of this review, all features in ResearchRabbit are free to use. The ResearchRabbit website insists that the tool will remain “free forever for researchers” [2].

## Comparison with Similar Products

Litmaps and Connected Papers are similar network map visualization tools which allow for the discovery of other related publications once a seed article is provided.

The key differences between these three tools are highlighted in Table 1.

**Table 1:** Comparison of ResearchRabbit, LitMaps and Connected Papers

Feature	ResearchRabbit	Litmaps	Connected Papers
Account requirements and cost	Account required No cost, all features are available for all users	Account required $120/year for Pro-version Pro-version allows for: Unlimited number of publications to be added for discovery maps Enables filter features of the discover maps, which allows for much more in depth analysis [3].	Without an account, users can create 2 maps per month (free) With an account, users can create 5 maps per month (free) $ 36.00/year for academics $120/year for businesses With paid accounts, users can have unlimited number of maps per month and have access to other features, such as saving newly discovered publications through the maps [4].
Number of seed publications supported	At least one, nomaximum number	Only supports one for a Seed map Maximum of 20 (in the free version) for Discover map	One
Types of maps that can be created	Network and timeline maps can be viewed by authors or publications Maps can toggle between “all citations”, “allreferences”, and “similar work”	Seed map-users add one seed publication. Litmaps will generate a map with suggested related publications. Discover map-users can add more than one seed publication enabling a more refined list of recommended publicationscompared to the seed map. Map view-users can create maps based on publications they have selected. No recommended publications are provided in this view. Map view allows users to see the connections just between their	Only one type of map is created based on the one seed publication. In a tabular format, users can also see publications that were most cited by the seed publication under “Prior works” as well as publications that cited the seed publication under “Derivative works”.This information can only be downloaded in BibTeX format.
selected publications.			
How are publications recommended?	Based on citations and AI	Based on citations of seed article	Co-citation and bibliographic coupling, meaning that if publications have overlapping citations and references, they are more likely to be recommended [4]

## Conclusion

ResearchRabbit offers an extensive range of functions, which can be overwhelming at first. However, once users overcome the learning curve, ResearchRabbit can become a powerful discovery tool for researchers doing unstructured searching. The various types of maps that can be generated and the different views available in each map cater to the needs of diverse users. Its use of AI makes the publication recommendations unique compared to its competitors and provides users with a more comprehensive set of recommended publications than users would get otherwise.
